# Substitution of the SEMA3A basic domain with the VEGF_165_ heparin-binding domain enhances anti-angiogenic potency *in ovo*

**DOI:** 10.1186/s40659-026-00684-z

**Published:** 2026-03-16

**Authors:** Indrė Valiulytė-Simaitė, Lina Šlekienė, Ingrida Balnytė, Viktorija Daukšaitė, Saulius Tacionis, Gabrielė Paškevičiūtė, Kristina Mašalaitė, Arūnas Kazlauskas

**Affiliations:** 1 Laboratory of Molecular Neurooncology, Neuroscience Institute, Lithuanian University of Health Sciences, Kaunas, LT- 50161 Lithuania; 2 Department of Histology and Embryology, Medical Academy, Lithuanian University of Health Sciences, Kaunas, LT-44307 Lithuania; 3 Faculty of Medicine, Lithuanian University of Health Sciences, Kaunas, LT-44307 Lithuania

**Keywords:** SEMA3A, VEGF, HBD domain, NRP1, Angiogenesis, Invasion, Proliferation, CAM

## Abstract

**Background:**

Angiogenesis plays a critical role in tumor development, with neuropilin receptors (NRPs) involved in this process through their interactions with vascular endothelial growth factor (VEGF) and semaphorins. SEMA3A, a member of class 3 semaphorins, demonstrates strong antiangiogenic potential. In this study, the C-terminal basic domain of SEMA3A was replaced with the heparin-binding domain (HBD) of VEGF_165_, hypothesizing that the resultant chimeric protein SEMA-PSI-IG-HBD would enhance antiangiogenic efficacy by: (1) competing with VEGF for NRPs (2) utilizing the HBD as a molecular anchor to increase the residence time and local concentration of the hybrid within the tumor microenvironment.

**Methods:**

To assess the effects of SEMA-PSI-IG-HBD on angiogenesis, a series of *in vitro* assays including microcapillary network formation, 3D cell spheroid sprouting, proliferation and interaction with NRP1 were carried out, whereas *in ovo* experiments were performed by using the chicken embryo chorioallantoic membrane (CAM) model.

**Results:**

Pull-down assays confirmed that the SEMA-PSI-IG-HBD hybrid specifically interacted with secreted NRP1 in a manner similar to the wild-type SEMA3A, validating the structural integrity of the chimeric protein’s receptor-binding interface. SEMA-PSI-IG-HBD and SEMA3A showed comparable inhibition in 2D microcapillary formation and 3D spheroid sprouting assays. However, the hybrid demonstrated markedly superior anti-angiogenic activity in the complex 3D environment of the CAM model.

**Conclusions:**

Our findings demonstrate that by combining competitive occupancy of NRP1 with potent anti-angiogenic signaling, SEMA-PSI-IG-HBD represents a promising therapeutic candidate for the regulation of pathological vascularization in cancer.

**Supplementary Information:**

The online version contains supplementary material available at 10.1186/s40659-026-00684-z.

## Background

Angiogenesis is one of the key processes of tumor development and maintenance, as the formation of new blood vessels is required to provide nutrients and oxygen to the tissue [1]. Therefore, antiangiogenic molecules are of immense importance in the search for anticancer drugs. The most used antiangiogenetic agents target vascular endothelial growth factor (VEGF) [2, 3]. Although anti-VEGF therapy improves survival in most cancer patients, some patients experience little or no benefit due to drug toxicity and various adverse side effects, such as hypertension, arterial thromboembolic events, and complications in wound healing [4]. Consequently, the need to develop more effective antiangiogenic molecules for cancer treatment remains relevant.

Semaphorins are a large family of proteins widely expressed in various tissues, particularly in the central nervous system during its development. They participate in organ development, tissue repair, immune responses, metabolic disorders, and tumorigenesis [5–8]. Based on the protein structure and phylogenetic analyses, semaphorins are divided into eight subclasses. Class 1 and Class 2 proteins are found in invertebrates, Classes 3, 4 and 7 – in vertebrates, Class V – in viruses, and only Class 5 semaphorins are found in both vertebrates and invertebrates [9, 10]. Recent studies have demonstrated that secreted class 3 semaphorin proteins (SEMA3s), which consist of 7 members SEMA3(A-G), are associated with cancer cell proliferation, invasiveness, and metastatic spread, making them promising targets for cancer treatment [11, 12]. The biological activity of SEMA3 proteins occurs on the surface of effector cells through the formation of the ternary semaphorin-neuropilin-plexin complex. This interaction involves several SEMA3 domains: the N-terminus, a ~ 500-amino-acid-long SEMA domain that is highly conserved across species; the cysteine-enriched plexin-semaphorin-integrin (PSI) motif, essential for forming the homodimer structure and facilitating ligand-receptor binding; and the C-terminus, which includes an immunoglobulin-like (Ig-like) domain along with a basic domain featuring disulfide bridges that contribute to SEMA3 dimerization and receptor binding [10, 6]. Depending on the interaction of SEMA3 with receptors or the proteolytic cleavage of proteins, these interactions can mediate either tumor-promoting or tumor-suppressing functions [13–16]. Nearly all SEMA3 family members were shown to be important regulators of angiogenesis processes [17]. Importantly, the VEGF protein, which plays a vital role in tumor angiogenesis, interacts with the same SEMA3 co-receptor, neuropilin [18].

Neuropilin receptors (NRPs: NRP1 and NRP2) are highly expressed in various cell types, including endothelial and cancer cells. They cooperate with other receptors (plexins, integrins, cadherins, and VEGF receptors) to initiate downstream signaling pathways and regulate processes, such as migration, proliferation, invasion, and angiogenesis [19, 20]. The N-terminal SEMA domain of semaphorins is crucial for binding to the a1a2 region located at the N-terminus of NRPs. The non-conservative C-terminal regions of semaphorins contain furin cleavage sites (RXXR or KXRXRR) that, upon the C-terminal arginine unmasking by cleavage, are important for SEMA3 interaction with the b1b2 region of NRPs, where the arginine-binding cleft of the b1 domain plays a critical role [21–24]. Noteworthy, the b1b2 domain of NRPs also interacts with VEGF via its heparin-binding domain (HBD) [25–27]. Therefore, a direct competition of semaphorins with VEGF for binding to NRPs provides a mechanism of angiogenesis inhibition [22, 23, 28, 29].

**Fig. 1:**
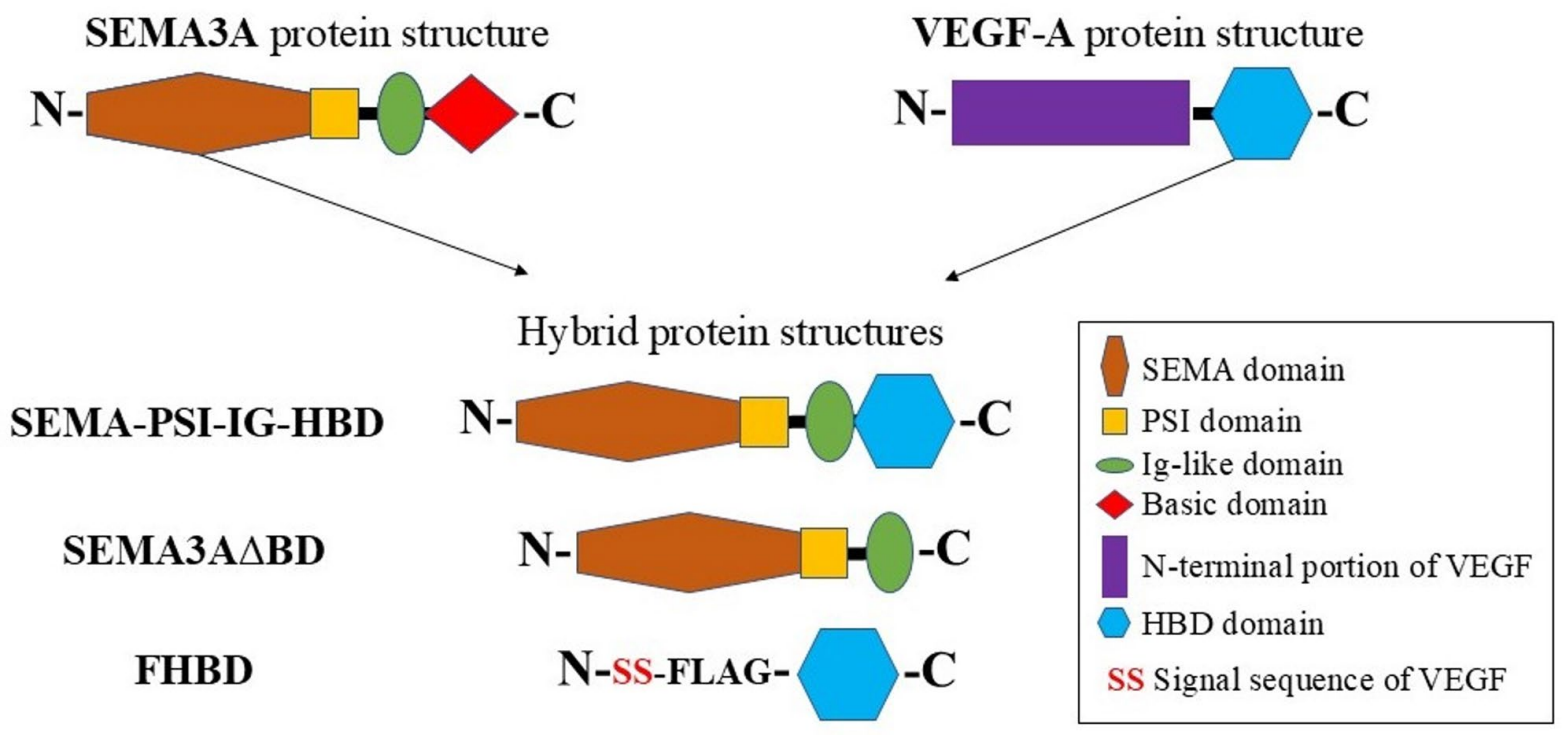
Construction of hybrid proteins. The structure of the SEMA3A protein consists of the SEMA domain, a PSI (plexin-semaphorin-integrin) domain, an Ig-like (immunoglobulin) domain, and a basic domain. The structure of the VEGF-A protein structure includes the N-terminal portion of VEGF and a heparin-binding domain (HBD). SEMA3A lacking the C-terminal basic domain denoted as SEMA3A∆BD. SS represents the signal sequence and F indicates the flag

In this context, the idea of the study was to generate a chimeric derivative of the semaphorin SEMA3A and VEGF (SEMA-PSI-IG-HBD) that would be expected to demonstrate stronger antiangiogenic potential compared to native SEMA3A, the inhibitory function of which on tumor growth and angiogenesis have been reported for different types of cancers [17]. To achieve this goal, the C-terminal basic domain of SEMA3A was substituted with the HBD of VEGF_165_ (See Fig. 1). The rationale was twofold: first, to exploit the structural conservation of the C-terminal arginine motif (the ‘CendR’ rule) to ensure superior competitive docking against endogenous VEGF_165_ for the NRP1 receptor; and second, to utilize the HBD as a molecular anchor [30, 31] to increase the residence time and local concentration of the inhibitory Sema3A ‘warhead’ within the rich heparan sulfate proteoglycan (HSPG) environment of the tumor microenvironment. The effects of the SEMA-HBD hybrid on angiogenesis were tested *in vitro* by examining endothelial cell sprouting, the ability to form microcapillary structures, and cell proliferation, whereas *in vivo* (*in ovo*) experiments were performed by using the chicken embryo chorioallantoic membrane (CAM) model.

## Methods

### Cell lines and transfection

Human embryonic kidney cells 293 FT (Invitrogen, R70007) were grown in Dulbecco’s Modified Eagle Medium (DMEM)/F-12 and GlutaMAX medium (Gibco, 10565018), supplemented with 10% fetal bovine serum (FBS; Gibco, 10500064), 100 IU/mL of penicillin, and 100 µg/mL of streptomycin (p/s; Gibco, 15140122).

Human umbilical vein endothelial cells (HUVECs; Gibco, C-003–5 C) were obtained as cryopreserved primary cells from a single human umbilical cord donor. According to the Certificate of Analysis for the lot used, these cells are characterized by positive immunofluorescence for endothelial markers CD31 and von Willebrand factor, negative for α-actin, and demonstrate functional uptake of DiI-Ac-LDL, confirming their endothelial identity and purity. HUVECs were grown in endothelial growth cell Medium 200PRF (Gibco, M200PRF500), supplemented with 1 × Low Serum Growth Supplement (LSGS; Gibco, CS00310) and 1 × p/s. Cells were used for experiments until passage eight.

Stable glioblastoma cell lines U87 MG expressing SEMA3A, SEMA-PSI-IG-HBD and control protein VENUS were generated by transfecting the cells with corresponding vectors (described below) pTO/SEMA3A-IRES2-VENUS, pTO/SEMA-PSI-IG-HBD-IRES2-VENUS and pTO/IRES2-VENUS and performing selection with zeocine as described in [32]. The stable U87 cells were grown in a high glucose DMEM supplemented with GlutaMAX (Gibco, 61965059). All cell lines were incubated in a humidified atmosphere of 95% air and 5% CO_2_ at 37 °C.

Expression vectors were delivered into 293 FT cells using Lipofectamine 2000 Transfection Reagent (Invitrogen, 11668027), according to the manufacturer’s instructions. The successful insertion of the plasmids was confirmed by fluorescent microscope (Lumascope LS620, Etaluma, Carlsbad, USA) and reverse-transcription PCR assay (see Additional file 1, Figure S1 and Table S1 “Verification of expression vectors“).

### Construction of eucaryotic expression vectors

Bicistronic expression vectors pTO/SEMA3A-IRES2-VENUS encoding wild-type human SEMA3A and fluorescent protein VENUS (separated by the IRES2 element) and pTO/IRES2-VENUS encoding expression VENUS protein only were described in previous publication [33].

The bicistronic vector pTO/VEGF-IRES2-VENUS encoding human VEGF_165_ and VENUS proteins was constructed in two steps. First, DNA fragment coding VEGF_165_ with the frame-shift error at position 499 of the protein-coding nucleotide sequence was generated by PCR using primers 5’-GGGGATCCATGAACTTTCTGCTGTCTTG-3’ and 5’-GTGACAAGCCGAGGCGGTGAAGATCTAA-3’ and template BC058855 purchased from Dharmacon Inc. (cat. No. MHS1010-202746539, clone ID: 5313912). The PCR product was digested with BamHI and BglII and ligated to pTO/IRES2-VENUS opened with BamHI. This resulted in intermediate construction pTO/VEGF-fs499A-IRES2-VENUS. In the second step, the frame-shift error of VEGF_165_-coding sequence was corrected by using Phusion Site-Directed Mutagenesis Kit (ThermoFisher Scientific), primers: 5’-TTCCTGCAAAAACACAGACTCGCG-3’ and 5’-CATTTACACGTCTGCGGATCTTG-3’ and pTO/VEGF-fs499A-IRES2-VENUS as a template. The integrity of VEGF_165_-coding sequence was confirmed by sequencing.

The bicistronic vector pTO/FHBD-IRES2-VENUS encodes recombinant protein FHBD consisting of signal sequence of VEGF_165_ (aa 1–27), FLAG peptide and HBD domain of VEGF_165_ (aa 142–191) and fluorescent protein VENUS (separated by the IRES2 element) was constructed by replacing the region of VEGF_165_ between the signal sequence and HBD domain (aa 28–141) with the FLAG peptide sequence. This was achieved by using Phusion Site-Directed Mutagenesis Kit (ThermoFisher Scientific), primers: 5’-GACTACAAGGACGATGATGACAAGCCCTGTGGGCCTTGCTCAGAG-3’ and 5’-TGCAGCCTGGGACCACTTGG-3’, and vector pTO/VEGF-IRES2-VENUS as a template. The integrity of FHBD-coding sequence was confirmed by sequencing.

The bicistronic vector pTO/SEMA-PSI-IG-HBD-IRES2-VENUS encoding recombinant protein SEMA-PSI-IG-HBD and fluorescent protein VENUS (separated by the IRES2 element) was constructed in two steps. First, an intermediate vector pTO/SS-Nhe-HBD was generated by replacing the region of VEGF_165_ between the signal sequence and HBD domain (aa 28–141) with the NheI restriction site by using Phusion Site-Directed Mutagenesis Kit (ThermoFisher Scientific), primers: 5’-GCTAGCCCCTGTGGGCCTTGCTCAGAG-3’ and 5’-TGCAGCCTGGGACCACTTGG-3’, and vector pTO/VEGF-IRES2-VENUS as a template. In the second step, SEMA-PSI-IG-coding DNA fragment was generated by PCR using primers 5’-TCGGATCCATGGGCTGGTTAACTAGGATTG-3’ and 5’-GATGCTAGCTGTGTCAATGACTTCCAGGGT-3’ and a template BC111416 purchased from Dharmacon Inc. (cat. No. MHS6278-211690268, clone ID: 40034963). The PCR product was digested with BamHI and NheI and ligated to pTO/SS-Nhe-HBD opened with BamHI and NheI.

SEMA3A∆BD coding vector pTO/SEMA3A∆BD was constructed by removing HBD-IRES2-VENUS part from the vector pTO/SEMA-PSI-IG-HBD-IRES2-VENUS by digestion with NheI and XbaI and closing vector with ligation.

Biotin acceptor domain-coding vector pEBB/PP [34] and ER-localized BirA coding vector pEBB/BirA-ER were kindly provided by prof. Kalle Saksela (Department of Virology, University of Helsinki).

Vector pTO/SSBAD-SEMA3A was constructed in two steps. First, the intermediate vector pTO/SSBAD coding signal sequence of VEGF (SS, aa 1–27) fused to the biotin acceptor domain (BAD) was created. For this purpose, BAD-coding DNA fragment was generated by PCR using primers 5’-TTTGCTAGCGGTAAGGCCGGAGAGGG-3’, 5’-CCCGAATTCCCCGATCTTGATGAGACCCTGAC-3’ and pEBB/PP vector as a template. The PCR product was digested with NheI and EcoRI and ligated to pTO/SS-Nhe-HBD opened with NheI and EcoRI, thereby replacing HBD with BAD sequence. In the second step, the DNA fragment coding SEMA3A without signal sequence was generated by PCR using primers 5’-CCCGAATTCAAGAACAATGTGCCAAGGCTG-3’, 5’-TTTTCTAGATCAGACACTCCTGGGTGCCCTC-3’ and BC111416 (see above) as a template. The PCR product was digested with EcoRI and XbaI and ligated to pTO/SSBAD opened with EcoRI and XbaI.

Vector pTO/SSBAD-SEMA3A∆BD was constructed by generating DNA fragment coding SEMA3A without signal sequence and basic domain using primers 5’-CCCGAATTCAAGAACAATGTGCCAAGGCTG-3’, 5’-GATGCTAGCTGTGTCAATGACTTCCAGGGT-3’ and BC111416 (see above) as a template. The PCR product was digested with EcoRI and NheI and ligated to pTO/SSBAD opened with EcoRI and XbaI.

Vector pTO/SSBAD-SEMA-PSI-IG-HBD was constructed by generating DNA fragment coding SEMA-PSI-IG-HBD using primers 5’-CCCGAATTCAAGAACAATGTGCCAAGGCTG-3’, 5’-CAATCTAGATCACCGCCTCGGCTTGTCAC-3’ and pTO/SEMA-PSI-IG-HBD-IRES2-VENUS as a template. The PCR product was digested with EcoRI and XbaI and ligated to pTO/SSBAD opened with EcoRI and XbaI.

Vector pTO/sNRP1-FLAG coding FLAG-tagged soluble NRP1 [35] was constructed by 3-step PCR using direct primer 5-AAGGATCCACCATGGAGAGGGGGCTG-3’ (steps 1–3), reverse primers 5’-GTCCTTGTAGTCTTTGATACCTGATTGTATGG-3’ (step 1), 5’-CTTGTCATCATCGTCCTTGTAGTC-3’ (step 2), 5’-GGGGAATTCACTTGTCATCATCGTCCTTGTAG-3’ (step 3) and a template BC007533 (in the 1-st step) purchased from Dharmacon Inc. (cat. No. MHS6278-202826331, clone ID: 2958475). The final PCR product was digested with BamHI and EcoRI and ligated to pcDNA4/TO (Invitrogen) opened with BamHI and EcoRI.

### Co-immunoprecipitation and Western blot analysis

HEK393FT cells, seeded onto 22 cm^2^ Petri plates, were transfected with 3 plasmids in each plate – (1) sNRP1-FLAG coding plasmid or VENUS-expressing plasmid (control), (2) BirA-ER-coding plasmid, and (3) one of the semaphorin protein-coding plasmids (SEMA3A, SEMA-PSI-IG-HBD, and SEMA3A∆BD) – by using jetOPTIMUS transfection reagent (Polyplus). On the third day after transfection, the medium was collected, supplemented with the Halt Protease and Phosphatase Inhibitor Cocktail (Thermo Fisher Scientific Inc.) and cleared by centrifugation at 1000 g / 4℃ / 5 min. Next, 1.8 ml of each media sample was transferred to a 2 ml centrifuge tube and supplemented with 1.5 µg of FLAG-specific antibody (cat. No. MA1-91878, Thermo Fisher Scientific Inc.) and 30 µl of Dynabeads Protein A paramagnetic bead (Thermo Fisher Scientific Inc., cat. No. 10001D) slurry. Samples were incubated for 2 h, at 4℃ under slow rotation followed by bead wash 3 times with PBS supplemented with 0,05% Tween-20 (PBS-T). After the last washing step, the beads were resuspended into 4x Laemmli buffer and protein complexes were denatured by heating samples at 95℃ for 5 min.

For whole-cell extract (WCE) preparation, transfected cells were lysed in RIPA lysis buffer (50 mM TrisHCl (pH 7.5), 150 mM NaCl, 1% Igepal CA-630, 0.5% sodium deoxycholate, 0.1% SDS) supplemented with the Halt Protease and Phosphatase Inhibitor Cocktail (Thermo Fisher Scientific Inc. cat. No. 78440) and centrifuged 40 min at 12.000 × g at 4 °C. Supernatants were collected, aliquots of 60 µg were mixed with 4x Laemmli buffer and denatured by heating at 95℃ for 5 min and stored at -80℃ before use. For Western blot analysis, samples were loaded onto 7.5% SDS-PAGE and transferred to nitrocellulose membrane. The membrane was blocked with 10% non-fat milk in PBS overnight. For detection of biotinylated proteins, the membranes were incubated with horseradish peroxidase- (HRP-) Streptavidin (Invitrogen, cat. No. 43-4323, dilution 1:2000 in 5% BSA/PBS) at 25 °C for 1 h followed by washing with PBS-T before protein signal detection. For detection of sNRP1-FLAG, the membrane was incubated with FLAG-specific rabbit polyclonal antibody (cat. No. MA1-91878, Thermo Fisher Scientific Inc. dilution 1:500) at 25 °C for 3 h. After washing with PBS-T, the membrane was incubated with the secondary HRP-conjugated goat anti-rabbit antibody (Invitrogen, cat. No. 65-6120, dilution 1:2000) for 40 min at 25 °C. For detection of β-actin, membrane was incubated with FLAG-specific rabbit polyclonal antibody (cat. No. MA1-91878, Thermo Fisher Scientific Inc. dilution 1:500) at ambient temperature for 3 h. After washing with PBS-T, the membrane was incubated with the secondary HRP-conjugated goat anti-rabbit antibody (Invitrogen, cat. No. 65-6120, dilution 1:2000) for 40 min at 25 °C. For detection of β-actin, membrane was incubated with the primary monoclonal mouse antibody against bet-actin (Antibodies-Online, cat. No. ABIN559692, dilution 1:2000) for 1 h at 25 °C followed by incubation with the HRP-conjugated anti-mouse secondary antibody (Life Technologies, catalog No. 626520, dilution 1:2000) for 40 min at 25 °C. Protein signals in all antibody-treated membranes were visualized using TMB substrate (3,3ʹ,5,5ʹ-Tetramethylbenzidine, Merck, cat. No. T0565) and captured with the digital scanner.

### Endothelial tube formation assay

The 293 FT cells were transfected with constructed expression vectors and, after 24 h, transferred to the round-bottomed wells of the Nunclon Sphera 96U Bottom Plate to generate cell spheroids (5 × 10^3^ cells per spheroid). The next day, a 24-well plate was covered with 40 µl of Geltrex (Gibco, A1413201) and incubated at 37 °C for 30 min to solidify. Cell spheroids were collected and placed on top of the Geltrex (6 spheroids per well, with at least 3 replicates per group). The plate was incubated overnight at a 45° angle to aggregate the spheroids in one corner of the well, ensuring that the spheroids would not hinder endothelial cells from forming a microcapillary network. Finally, 5 × 10^4^ HUVECs were suspended in media from cell spheroids and seeded on top of the Geltrex. After 6 h, fully formed microcapillary networks were captured (from 3 different areas) with a microscope at 10X magnification and analyzed using the Fiji angiogenesis analyzer program [36]. The number of meshes, mean mesh area, number of junctions, master segments, total microcapillary network length, and number of isolated segments of the microcapillary network were evaluated.

The coating volume, polymerization conditions, and HUVEC seeding density were selected based on manufacturer recommendations and commonly used angiogenesis assay protocols and were kept constant across all experiments to ensure reproducibility.

### 3D sprouting assay

HUVECs were seeded in round-bottomed wells (1 × 10^3^ cells/well) of the Nunclon Sphera 96U Bottom Plate and incubated in M200 media, supplemented with 1 × LSGS and p/s, for 24 h to form cell spheroids. The spheroids were then collected and centrifuged at 210 × g for 4 min at room temperature. The supernatant was removed, and the spheroids were suspended in ice-cold 1.5-mg/mL Collagen I solution (Gibco, A1064401) with 10% of FBS, pH = 7.2, and seeded onto a 24-well plate (10 spheroids/well, per group). The collagen mixture was prepared according to the manufacturer’s instructions and maintained on ice prior to polymerization. The pH of the prepared collagen solution was verified using pH indicator paper by testing a small aliquot to avoid contamination of the bulk solution and, when necessary, adjusted to pH 7.2 using sterile NaOH under aseptic conditions. The spheroid–collagen mixture was seeded into a 24-well plate (10 spheroids per well per group) and incubated at 37 °C for 30 min to allow collagen polymerization. Media, as a source of hybrid proteins, were collected from transfected 293FT cells, centrifuged at 180 × g for 5 min at room temperature, and placed on top of the collagen-containing cell spheroids. The cell spheroids were captured immediately with a microscope (0 point) and after 16 h. The Fiji program [36] was used to analyze the spheroid area expansion which was calculated as the difference between the total spheroid area at 16 h and the initial spheroid area at 0 h. For presentation, area expansion values were normalized to the control group, which was set to 100%. (the difference between the spheroid area at 16 h and 0 h).

### Proliferation assay

HUVECs were seeded in flat-bottomed wells (1 × 10^4^ cells/well) and incubated in M200 media, supplemented with 1 × LSGS and p/s. After 24 h, the media was changed to that from transfected 293FT cells, and HUVECs were incubated for 8 h. Then, the cells were incubated with 100 µl of 0.5 mg/mL MTT (3-(4,5-dimethylthiazol-2-yl)-2,5-diphenyltetrazolium bromide) reagent (Invitrogen, cat. No. M6494) for 3 h. The formed formazan crystals were dissolved with 100 µl of dimethyl sulfoxide (Sigma-Aldrich, cat. No. D8418). The optical density was measured at 550 nm and 620 nm using Multiskan GO Microplate Spectrophotometer (ThermoFisher Scientific).

### *In ovo* CAM model and study groups

Fertilized chicken eggs were acquired from a local hatchery and placed in an incubator (Maino incubators, Oltrona di San Mamette, Italy) at 37 °C temperature and 60% relative air humidity. An automatic rotator was used for egg rolling once per hour until day 3 of embryo development (EDD3). At EDD3 the automatic rotation was switched off, eggshells were cleansed with prewarmed 70% ethanol, and a small hole was drilled in the location of an air chamber. According to the size of an egg, approximately 2 mL of albumin was withdrawn with a sterile syringe to detach the CAM from the shell. After that, a small square of approximately 1 cm^2^ was drilled, the shell of an egg was carefully removed, and a created window was sealed with sterile cellophane tape. The eggs were placed back into the incubator until further placement of tumor cells on the CAM at EDD7. The following 3 study groups were investigated: (1) VENUS (*n* = 25), (2) SEMA-PSI-IG-HBD (*n* = 22), (3) SEMA3A (*n* = 21).

### The placement of VENUS-, SEMA-PSI-IG-HBD- and SEMA3A-expressing cells onto the CAM

The number of 1 × 10^6^ cells was resuspended in 10 µL of serum-free DMEM and 10 µL of type I rat tail collagen (Gibco, Gaithersburg, MD, USA). In total, 20 µL of cell suspension were placed on absorbable surgical sponge (Surgispon, Aegis Lifesciences, India) which was cut manually with a blade to form pieces of 9 mm^3^ (3 × 3 × 1 mm). A 20 µL liquid mixture of tumor cells was gently pipetted and placed onto a piece of a surgical sponge. The cell suspension soaked in a sponge was placed onto the CAM among major blood vessels. At EDD12, after 5 days of incubation, the specimens were dissected together with CAM, fixed in a buffered 10% formalin solution for 24 h and embedded into the paraffin wax. The specimens were cut into 3 μm thickness sections using a microtome (Leica Microsystems Inc., Buffalo Grove, IL, USA), then stained with hematoxylin and eosin (H–E), and mounted with a mounting media (Roti HistoKit II, Carl Roth GbmH, Karlsruhe, Germany).

### Biomicroscopy *in vivo* and histomorphometric assay

Cell tumors from all study groups grafted on CAMs of chick embryos were captured *in ovo* at EDD12 (5 days post grafting) using an Olympus stereomicroscope SZX2-RFA16 (Olympus Corporation), supplied with a digital Olympus camera DP72 (Olympus Corporation, Tokyo, Japan). Examination of histological slides stained with H–E was performed using an Olympus light microscope BX53F (Olympus Corporation) equipped with a digital EXi Aqua camera (Canada). Histological images of tumors on CAM were acquired using image analysis software Image-Pro^®^ Plus (version 7.0, Media Cybernetics, USA). The number of blood vessels in all study groups was evaluated by capturing every H–E-stained CAM at 4x magnification directly under the grafted tumor. All blood vessels bigger than 10 μm were counted in the CAM of the same length (1600 μm).

### Statistical analysis

The normality of the data was checked using the Shapiro–Wilk test. The parameters of microcapillary structures and 3D sprouting were presented as means ± standard deviation. Differences between the VENUS, SEMA3A, VEGF, SEMA-PSI-IG-HBD, SEMA3A∆BD, and FHBD groups were compared using one-way ANOVA with Tukey`s correction. The significance level was set at *p* ≤ 0.05. Experiments were independently repeated at least three times.

## Results

### Effect of SEMA3A-VEGF hybrid on endothelial cell invasion

**Fig. 2:**
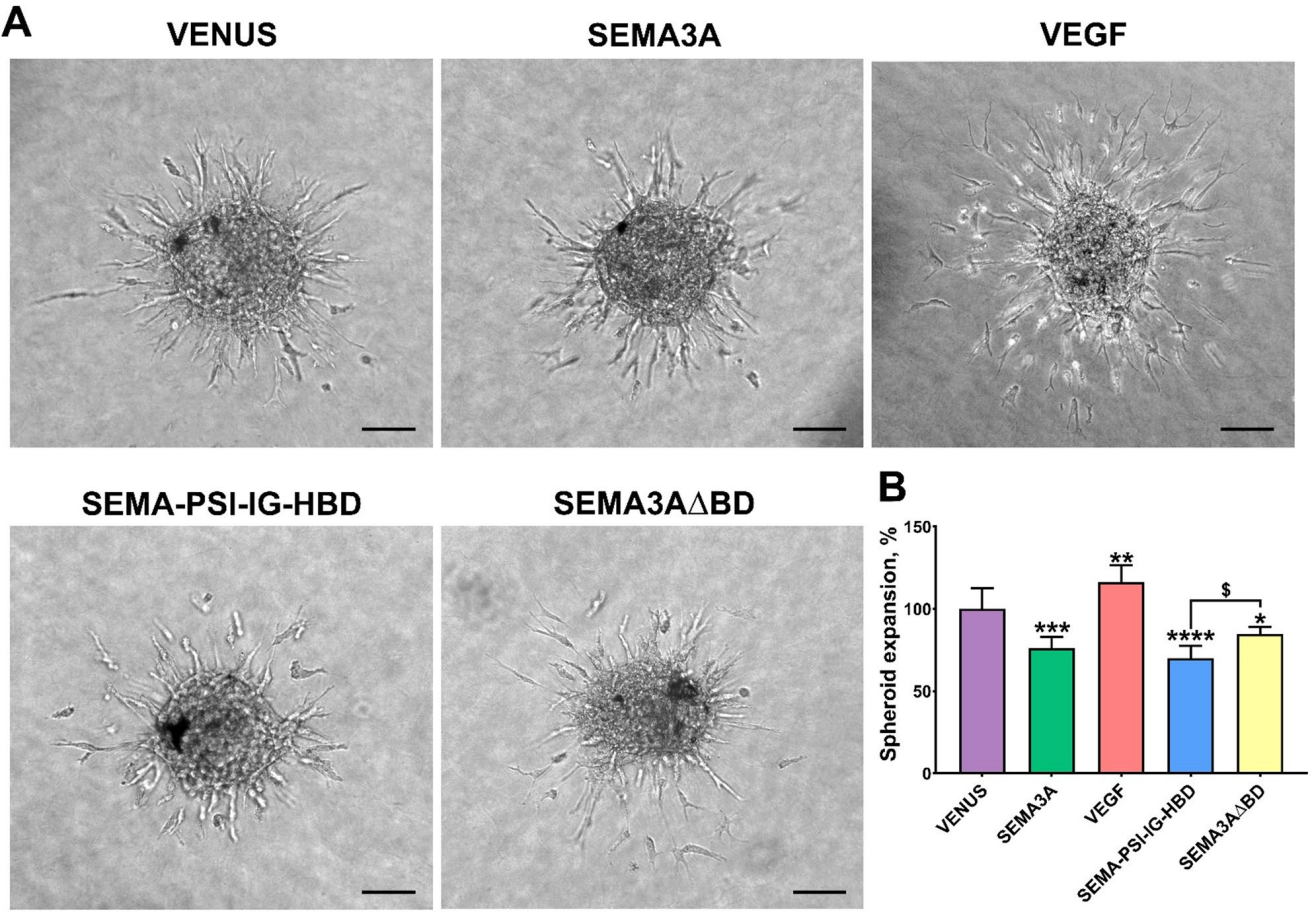
Effects of SEMA3A-VEGF hybrid on endothelial cell invasion. **A** Representative bright field microscopy images of HUVEC spheroids under different treatments, as indicated. Scale bar, 100 μm. **B** The spread of spheroid cells is indicated as spheroid expansion, %. Data are presented as mean ± SD. * – compared to the control VENUS group. *^$^*p ≤ 0.05* **p *≤ 0.01*, ***p *≤ 0.001*, and ****p *≤ 0.0001*

To characterize the effects of the hybrid SEMA3A-VEGF protein on the invasive phenotype of endothelial cells, spheroids of HUVECs were embedded in collagen and covered with media from transfected cells (as a source of our tested proteins). After 16 h of incubation, HUVECs spread widely around the spheroid in the control of the VENUS group. In all other groups, the spheroid expansion area was of a different extent (Fig. 2A). According to the statistical analysis (Fig. 2B), SEMA3A significantly suppressed (by 24.03%), while VEGF induced (by 16.4%) the formation of HUVEC branches (sprouts) around the spheroid periphery compared to the control VENUS (*p = 0.0003* and *p = 0.007*, respectively). The hybrid SEMA-PSI-IG-HBD protein exhibited the strongest inhibitory effect on HUVEC sprouting compared to VENUS (*p < 0.0001*): the spheroid expansion area was reduced by 30.2%. Importantly, the inhibitory effect of the semaphorin was abrogated in the SEMA3A∆BD group (*p < 0.04*), suggesting that the C- terminal basic domain of SEMA3A is necessary for its function.

### Effect of SEMA3A-VEGF hybrid on microcapillary formation

**Fig. 3:**
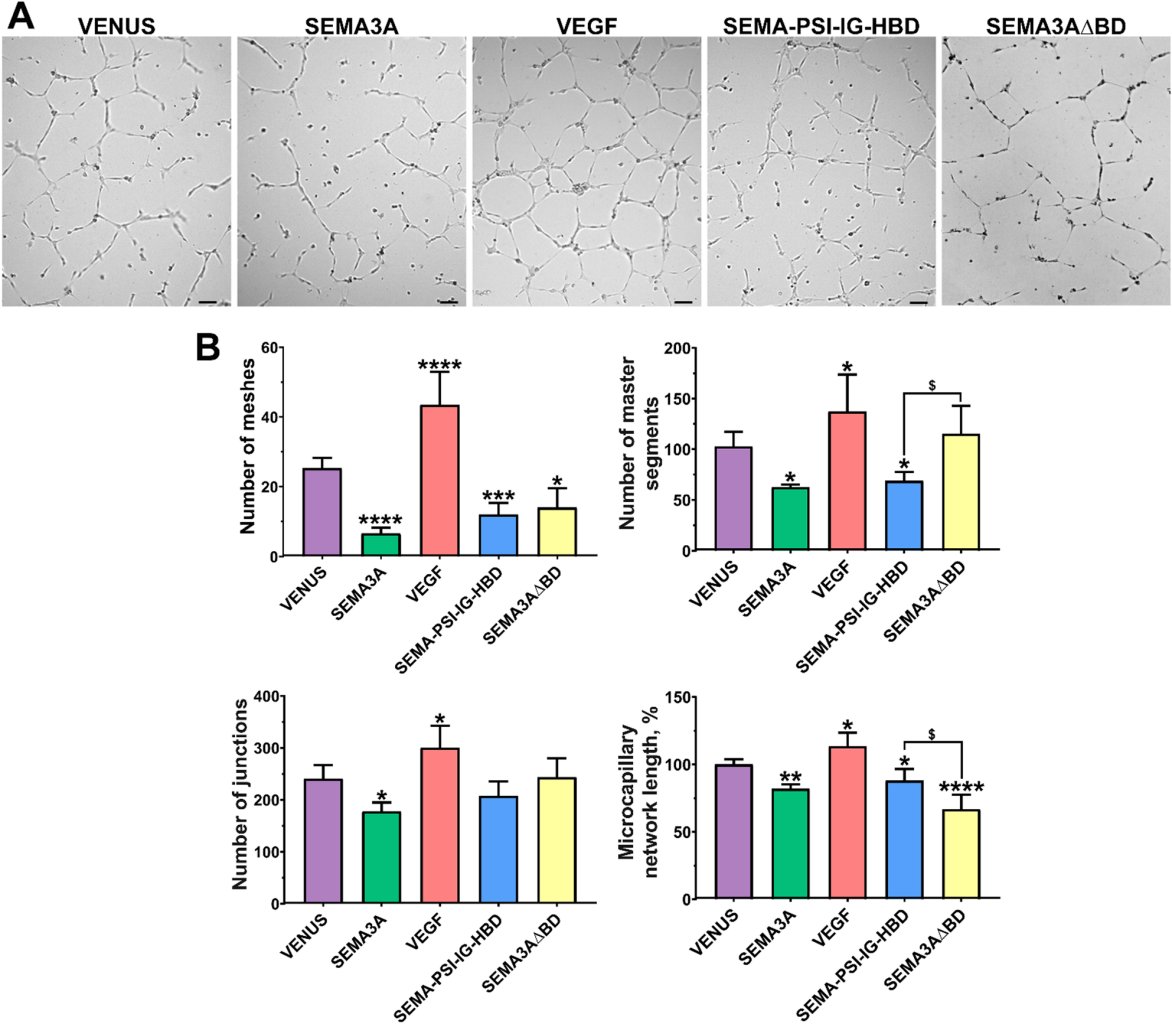
Effects of SEMA3A-VEGF hybrids on angiogenesis *in vitro*. **A** Representative bright field microscopy images of the HUVEC microcapillary network under different treatments, as indicated. Scale bar, 100 μm. **B** Statistical analysis of microcapillary tube formation. Data are presented as mean ± SD. * – compared to the control VENUS group. *^$^*p ≤ 0.05* **p *≤ 0.01*, ***p *≤ 0.001*, and ****p *≤ 0.0001*

Following the analysis of the 3D invasion assay, all hybrid molecules were further evaluated in an *in vitro* angiogenesis model. Endothelial cells were suspended in conditioned media from transfected cells and seeded onto Geltrex. After a 6-hour incubation, a fully formed microcapillary network of HUVECs was observed in the VENUS control group (Fig. 3A). In comparison, the antiangiogenic protein SEMA3A disintegrated the network by significantly reducing the mean number of meshes, master segments, and junctions, as well as the overall microcapillary length (Fig. 3B). In contrast, the proangiogenic VEGF significantly increased the microcapillary network’s length and density. Notably, the hybrid molecule SEMA-PSI-IG-HBD significantly suppressed the microcapillary network length by reducing the number of meshes and master segments. The differences between anti-angiogenic effects of SEMA3A and SEMA-PSI-IG-HBD were minor. The SEMA3AΔBD group exhibited a significantly higher number of segments compared to SEMA-PSI-IG-HBD (115.3 ± 27.57 vs. 68.75 ± 8.78, respectively; p < *0.018*), whereas the total network length was reduced to 66.83 ± 10.69 vs. 88.03 ± 8.54 (*p < 0.013*).

### Effect of SEMA3A-VEGF hybrid on proliferation of HUVECs

**Fig. 4:**
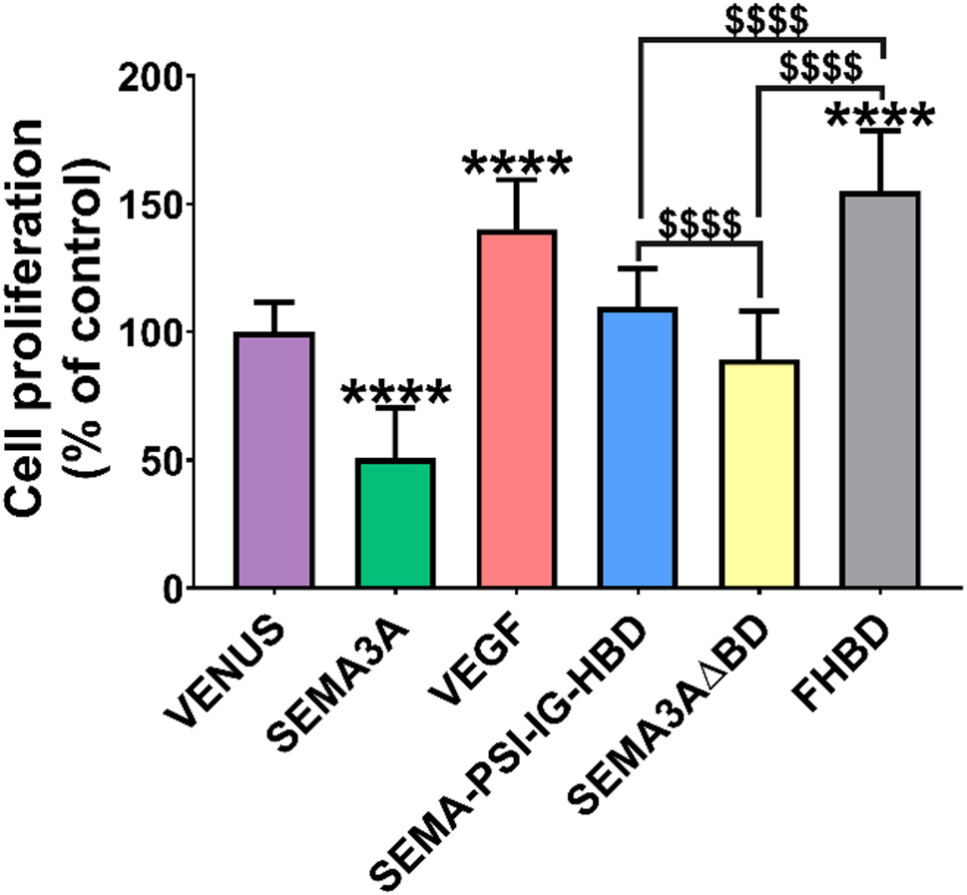
Effects of SEMA3A-VEGF hybrids on proliferation of HUVECs. Data are presented as mean ± SD. * – compared to the control VENUS group. ***p≤0.0001, p≤0.0001

To assess whether the SEMA3A-VEGF hybrid affects the proliferation of endothelial cells, an MTT assay was carried out. As shown in Fig. 4, SEMA3A significantly inhibited, while VEGF – promoted the growth of HUVECs by 49.23% and 39.93%, respectively, compared to the control VENUS (*p* < *0.0001*). However, the hybrid SEMA-PSI-IG-HBD molecule and SEMA3A∆BD showed no statistically significant effect on cell proliferation (*p = 0.117* and *p* = *0.066*, respectively). Notably, the FHBD domain alone enhanced endothelial cell proliferation by 54.99% compared to the VENUS control (*p* < *0.0001*). The proliferative effect induced by FHBD was comparable to that observed for VEGF, as no statistically significant difference was detected between these treatments (*p = 0.075*). Additionally, this domain is essential for the activity of the SEMA3A-VEGF hybrid, as cells demonstrated significantly greater proliferation when the domain was present in SEMA-PSI-IG-HBD compared to constructs lacking the C-terminal basic domain (*p < 0.0001*).

### Interaction between hybrid SEMA3A-VEGF protein and soluble NRP1

**Fig. 5:**
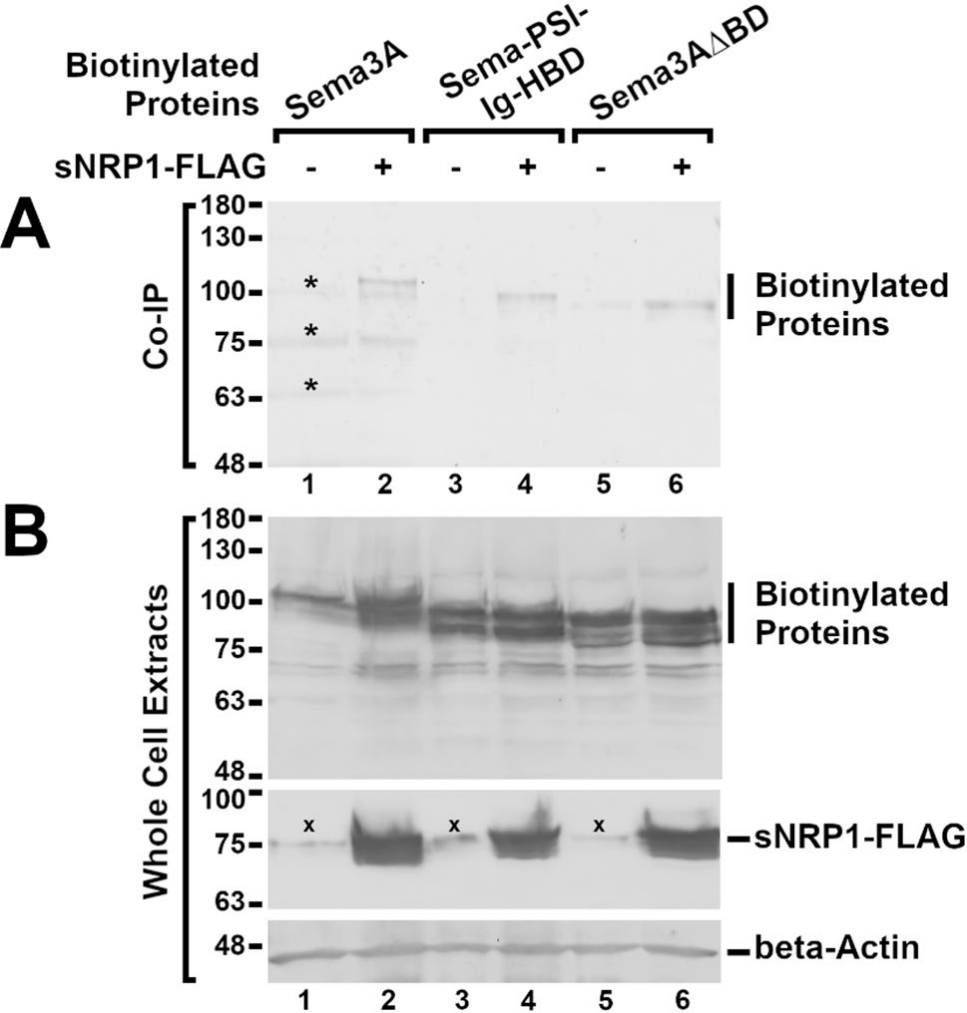
SEMA3A, SEMA-PSI-IG-HBD, and SEMA3A∆BD interacts with sNRP1. **A** Signal sequence-biotin acceptor domain-containing SEMA3A, SEMA-PSI-IG-HBD, and SEMA3A∆BD proteins biotinylated by co-expressed BirA-ER (Biotinylated Proteins) co-precipitated with NRP1-FLAG were detected by Western blot with Streptavidin-HRP. Bands that appeared on Co-IP membrane due to leakage of the protein standard to the lane 1 are indicated by asterisk (*) **B** Immunoblot with the use of Streptavidin-HRP was carried out on whole cell extracts to verify expression of biotinylated SEMA3A, SEMA-PSI-IG-HBD, and SEMA3A∆BD (Biotinylated Proteins) correspondingly to Co-IP samples indicated in panel A. Antibodies specific to FLAG and beta-actin were used to verify expression of sNRP1-FLAG and beta-actin in the same protein extracts. Unspecific protein signals indicated by symbol (×)

Protein complex co-immunoprecipitation was carried out to examine whether the hybrid SEMA-PSI-IG-HBD and the basic domain-lacking SEMA3A∆BD interact with NRP1 (see Fig. 5). SEMA3A, SEMA-PSI-IG-HBD, and SEMA3A∆BD were expressed in HEK293FT as biotinylated proteins together with or without (control) FLAG-tagged secreted form of NRP1 (sNRP1, see Materials and Methods). Protein complexes we precipitated from cell media with FLAG-specific antibodies whereas co-precipitated biotinylated proteins were identified by Western blot with Streptavidin-HRP. The result demonstrated that SEMA-PSI-IG-HBD specifically interacted with sNRP1 in a comparable manner to SEMA3A. Interestingly, the basic domain-lacking mutant of SEMA3A, SEMA3A∆BD, seems to also bind to NRP1. This indicates that the basic domain is not critically required for interaction between SEMA3A and NRP1, yet it plays a significant role for SEMA3A-regulated signaling.

### Effects of SEMA-PSI-IG-HBD on tumor vascularization

**Fig. 6:**
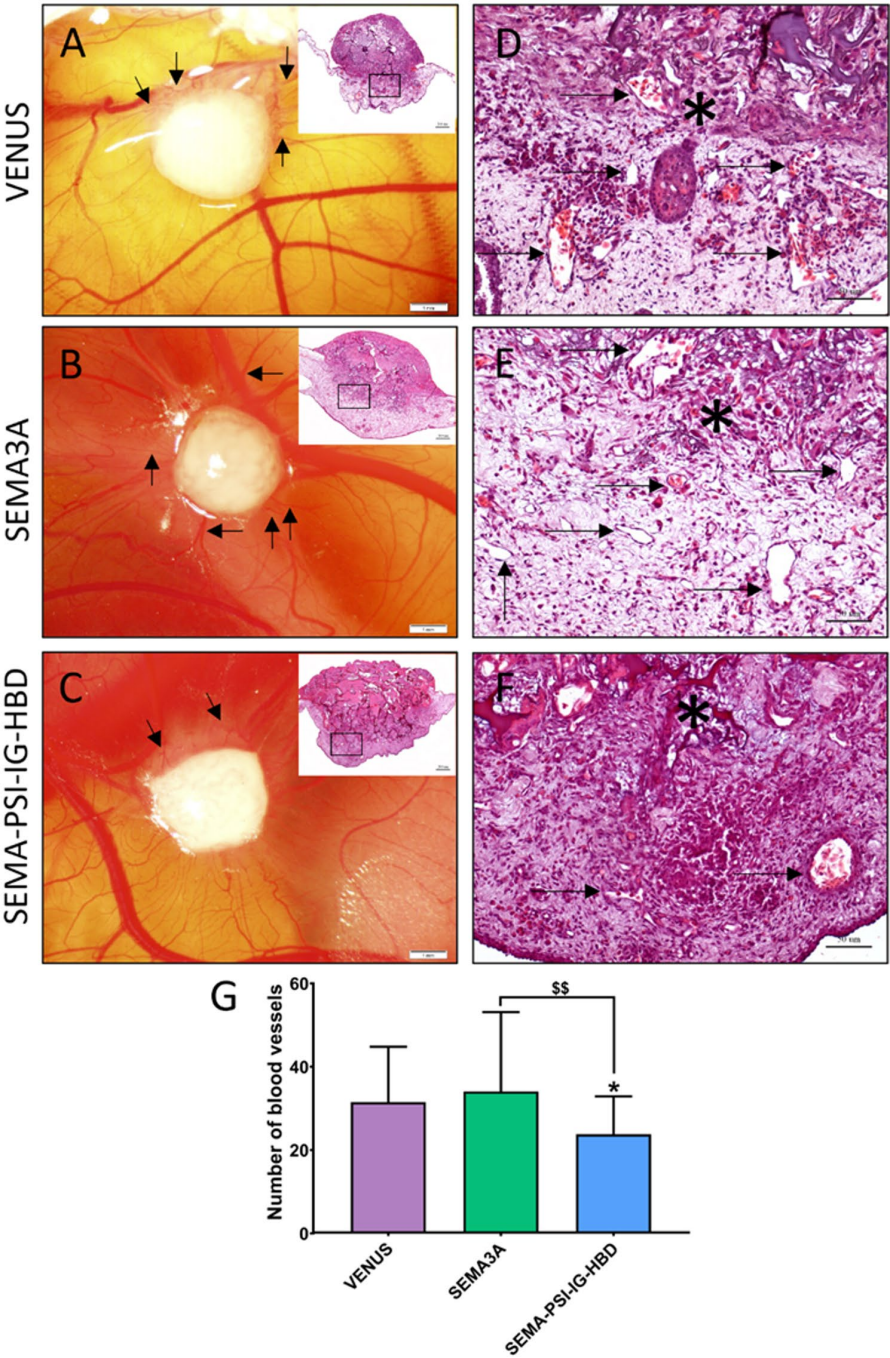
Biomicroscopy *in vivo* and microscopic images of the tumors on CAM. **A–C** pictures show tumor xenografts photographed on day 12 of embryo development via window in an eggshell. A small square in the histological image seen on the upper right corner of a macroscopic view indicates the magnified region seen in images D-F. **A** CAM with tumor formed from cells of VENUS group (*n* = 25). **B** CAM with tumor formed from cells of SEMA3A group (*n* = 21). **C** CAM with tumor formed from cells of SEMA-PSI-IG-HBD group (*n* = 22). **D–F** Hematoxylin and eosin staining show high magnification histological images of tumor invasion into the CAM on day 12 of embryo development from all study groups: Venus, SEMA3A and SEMA-PSI-IG-HBD respectively. Invasion into the CAM and damage to chorionic epithelium is clearly visible as there is no border between tumors and underlying mesenchyme (asterisk). **G** Statistical analysis of VENUS, SEMA3A and SEMA-PSI-IG-HBD on blood vessel formation in the CAMs mesenchyme. Data are presented as mean ± SD. *p < 0.05 – compared to the control VENUS group, ^$$^*p < 0.01*. ChE: Chorionic epithelium, BV: Blood vessel, M: Mesenchyme, and T: Tumor. Scale bars: **A**–**C**—1 mm and 200 μm (upper right images); **D**–**F**—50 μm

Tumor xenografts established on the CAM displayed distinct angiogenic profiles depending on U87 MG cell groups expressing different recombinant proteins (see Fig. 6). Tumors derived from control VENUS cells exhibited a dense network of small blood vessels around the neoplastic mass (Fig. 6A), while SEMA3A-expressing tumors showed a characteristic “spoked-wheel” distribution pattern (Fig. 6B). In contrast, tumors expressing the chimeric SEMA-PSI-IG-HBD protein attracted markedly fewer vessels (Fig. 6C). Histological examination (Figs. 6D–F) confirmed that all groups exhibited destruction of the chorionic epithelium and direct invasion into the underlying mesenchyme. However, the density of blood vessels within the mesenchyme differed visibly between experimental conditions.

Quantification and statistical analysis of mesenchymal blood vessels on day 12 revealed significant differences among groups (Fig. 6G). The SEMA-PSI-IG-HBD group exhibited the lowest vessel count (24 ± 9), showing a significant reduction compared with VENUS (31.52 ± 13, *p* *= 0.026*) and SEMA3A (34.07 ± 19, *p* *= 0.003*). Contrary to our expectations based on prior *in vitro* experiments, SEMA3A failed to induce a significant reduction in vascularization in the CAM model as compared to control VENUS (*p = 0.671*). Together, these findings demonstrate that the SEMA-PSI-IG-HBD construct exerts a potent anti-angiogenic effect *in vivo*, surpassing that of native SEMA3A.

## Discussion

Regarding cancer, NRPs have been shown to be upregulated in gastric, prostate, colorectal, lung, and other cancers, and their expression is significantly associated with tumor progression [19, 37, 38]. Consequently, molecules targeting NRPs could serve as promising tools to control tumor-associated angiogenesis [19, 20, 39]. NRPs can be targeted by monoclonal antibodies, various peptides, microRNAs, and other small molecular inhibitors [20, 40]. Antibodies that block VEGF/NRP interaction inhibit cancer cell growth, invasion, proliferation, and angiogenesis [41, 42]. Peptides that bind to the b1 domain of NRP1 block the interaction between VEGFA_165_ and its receptor, while short hairpin RNA-mediated depletion of NRP1 suppresses the growth of drug-resistant breast cancer [43, 44]. However, the therapeutic application of antibodies is often limited by their large size and complexity, while peptides can exhibit nonspecific cytolytic activity. For example, Tat-C-RP7, which suppresses activation of the VEGFR2-PLCγ-ERK1/2 pathway, inhibits HUVEC proliferation, microcapillary formation, and induces apoptosis. It also inhibits glioma tumor growth and blood vessel formation in a mice model [40]. However, it remains unclear whether the peptide selectively accumulates in glioma tissue or affects other tissues as well.

Moreover, in the context of anti-VEGF therapy, limited clinical durability and treatment-associated toxicity have been linked to incomplete suppression of angiogenic signaling and compensatory pathway activation [2–4]. While VEGF-neutralizing antibodies effectively block VEGF–VEGFR2 interaction, NRP1 can continue to stabilize receptor complexes and participate in alternative pro-survival signaling cascades [45, 46]. In addition, VEGF-independent NRP1 signaling may contribute to endothelial plasticity and adaptive vascular remodeling [44, 47]. These mechanisms highlight potential limitations of strategies targeting VEGF alone and provide a rationale for approaches capable of modulating multiple NRP-dependent interaction interfaces simultaneously. Therefore, combination therapies or molecules targeting multiple factors involved in angiogenesis regulation are employed to improve patient responses. For instance, a mixture of anti-VEGF and anti-NRP1 nanobodies has been shown to more effectively inhibit tube-like network formation and endothelial cell proliferation *in vitro* and in a CAM model compared to individual nanobodies [48]. Additionally, dual antibodies targeting both anti-VEGFA and anti-NRP1 significantly suppressed tumor growth and blood vessel formation in a pancreatic ductal adenocarcinoma xenograft model, as compared with bevacizumab [49]. Therefore, new ways to control tumor-associated angiogenesis are still being sought.

In this study, we designed a novel hybrid molecule using proteins naturally found in human cells and which was expected to have several properties, namely: the ability to compete with the proangiogenic factor VEGF, interact with NRPs, and activate signaling pathways that inhibit angiogenesis. Since NRPs serve as multifunctional receptors for both the VEGF and SEMA3 families, which participate in regulating angiogenesis, we decided to fuse the antiangiogenic SEMA3A molecule with the NRP-binding portion of VEGF_165_. Both SEMA3A and VEGF proteins were shown to compete for interaction with the same receptor, NRP1, but at different regions: the SEMA domain of SEMA3A interacts with the a1a2 domains of NRPs, while the HBD domain of VEGF – with b1b2 domains [18, 28]. In the newly constructed hybrid SEMA-PSI-IG-HBD, the SEMA3A molecule lacking the C-terminal basic domain was directly fused to the HBD domain of VEGF_165_. The rationale behind the construction of this chimeric protein was twofold: first, to exploit the structural conservation of the C-terminal arginine motif (the ‘CendR’ rule) to ensure superior competitive docking against endogenous VEGF_165_ for the NRP1 receptor; and second, to utilize the HBD as a molecular anchor to increase the residence time and local concentration of the inhibitory SEMA3A ‘warhead’ within the rich heparan sulfate proteoglycan (HSPG) environment of the tumor microenvironment. By simultaneously engaging both the SEMA-binding (a1a2) and VEGF-binding (b1b2) domains of NRP1 within a single engineered ligand, this dual-interface strategy was intended to provide broader modulation of NRP1-dependent signaling than VEGF-neutralizing approaches alone.

With co-immunoprecipitation experiments, we demonstrated that the SEMA-PSI-IG-HBD hybrid binds to the secreted form of NRP1 (sNRP1) to a similar extent to the full-length SEMA3A. Interestingly, we also detected an interaction between sNRP1 and SEMA3A lacking the C-terminal basic domain (SEMA3A∆BD), indicating that basic domain, although is important for the signal relay from SEMA3A to NRP1, is not critically required for the physical association of SEMA3A and NRP1 (Fig. 5).

The effects of SEMA-PSI-IG-HBD on angiogenesis were tested using a 3D invasion assay, which is more physiologically relevant than the 2D models, as well as a rapid and quantitative microcapillary formation assay. The novel hybrid protein SEMA-PSI-IG-HBD demonstrated significant inhibitory effects on the invasion and microcapillary formation of endothelial cells to a similar extent as SEMA3A (Figs. 2 and 3), although it did not affect their proliferation (Fig. 4). The hybrid’s ability to inhibit invasion and angiogenesis *in vitro* without affecting endothelial proliferation suggests a selective mechanism that primarily modulates cytoskeletal organization rather than inducing cell cytotoxicity. Such a selective mechanism could represent a therapeutic advantage, allowing for targeted suppression of pathological angiogenesis while preserving normal endothelial viability. Notably, the HBD domain alone enhanced endothelial cell proliferation, which was comparable to the full-length VEGF effects. Our findings are entirely consistent with the literature when the experimental context is considered. Purified HBD fragments have been widely reported to act as competitive antagonists that inhibit endothelial proliferation by preventing VEGF_165_/NRP1 complex formation [45, 50]. However, when the HBD is expressed within a complex microenvironment – specifically on a Geltrex basement membrane – its high affinity for heparan sulfate proteoglycans triggers the displacement and liberation of sequestered endogenous growth factors, as described by earlier studies [30, 51]. This transition from ‘antagonist’ to ‘niche modulator’ explains the diverging results between purified protein and cell-based expression systems. However, in the SEMA-PSI-IG-HBD hybrid, this effect is functionally neutralized. The high-affinity HBD ensures that the inhibitory Sema domain is held in immediate proximity to the NRP1/Plexin complex, overriding any baseline pro-survival signals by directly triggering endothelial repulsion and cytoskeletal collapse.

The CAM model provided further insight into the functional consequences of SEMA-PSI-IG-HBD hybrid. The protein exerted a markedly stronger anti-angiogenic effect than native SEMA3A and VENUS control. These findings suggest that engaging both the SEMA-binding (a1a2) and HBD-binding (b1b2) domains of NRP1 within a single ligand may confer an advantage over SEMA3A alone. This is consistent with observations by other studies that reported that targeting NRP1 can yield therapeutic benefit, with NRP1 antagonists showing efficacy even in early clinical studies [20]. The observation that SEMA3A did not reduce vascularization in the CAM model stands in contrast to our own *in vitro* findings and numerous reports describing SEMA3A as a potent inhibitor of endothelial motility [17, 52]. We propose that this discrepancy arises from the competitive dynamics between SEMA3A and the VEGF165 isoform secreted by U87 cells. Specifically, both factors compete for the b1b2 binding domain of the NRP1 co-receptor. In our model, the abundance of SEMA3A likely displaces the VEGF_165_ from NRP1 at the HBD-binding b1 cleft [53], disrupting the spatial gradients necessary for organized vascular sprouting. Rather than suppressing vessel growth, this displacement – coupled with SEMA3A’s known ability to induce VE-cadherin phosphorylation and subsequent internalization [47] and activate autocrine TGF-β signaling [54] – results in a phenotype of vascular destabilization and hyperpermeability. This creates a disorganized yet robust vascular network that supports enhanced tumor progression in the CAM environment. These results emphasize that in the glioblastoma microenvironment, SEMA3A functions more as a vascular remodeling agent than a classical angiogenesis inhibitor. The stark contrast between the pro-angiogenic effect of SEMA3A and the potent anti-angiogenic activity of SEMA-PSI-IG-HBD in the CAM model is a direct result of the HBD-mediated ‘anchoring’ effect. In the complex *in vivo* environment of the CAM, soluble SEMA3A is subject to rapid diffusion and competitive displacement by endogenous VEGF_165_. In contrast, the SEMA-PSI-IG-HBD hybrid utilizes its HBD domain to facilitate high-affinity docking to the NRP1 b1b2 pocket and the surrounding extracellular matrix [30, 55]. This site-directed antagonism ensures that the inhibitory SEMA3A signal is concentrated at the endothelial interface, successfully outcompeting VEGF_165_ and suppressing vessel recruitment.

The findings of this study should be seen considering several important questions that remained unanswered. First, the direct (physical) and functional competition with VEGF, which we expect from the SEMA-PSI-IG-HBD hybrid, has yet to be examined. Specifically, future biophysical characterization is required to determine if the HBD-fused SEMA3A chimera achieves a higher binding affinity for the NRP1 b1b2 pocket than native VEGF_165_ (Kd approx. 1 nM [53, 55]). We hypothesize that the HBD domain acts as a high-affinity ‘molecular anchor,’ not only occupying the NRP1 docking site but also creating steric hindrance that prevents the assembly of the pro-angiogenic VEGFR2/NRP1 holoreceptor complex. Furthermore, it remains to be elucidated whether the hybrid facilitates a ‘ligand-switching’ mechanism on the endothelial surface, where the local concentration of SEMA-PSI-IG-HBD in the Geltrex-rich extracellular matrix effectively displaces sequestered VEGF165 from heparan sulfate proteoglycans. This spatial competition moving from a pro-angiogenic matrix to an inhibitory matrix likely represents a critical component of the hybrid’s efficacy observed in our CAM models. Experimental validation using surface plasmon resonance or competitive ELISAs will be essential to confirm that the SEMA-PSI-IG-HBD hybrid functions as a true ‘dominant-negative’ ligand at the vascular-tumor interface.

The second issue we would like to address in our future studies involves the detailed characterization of the intracellular signaling cascades triggered by the SEMA-PSI-IG-HBD hybrid. Specifically, we aim to investigate the hybrid’s ability to modulate the VEGF-stimulated recruitment of the GIPC1/adaptor complex at the NRP1 cytoplasmic tail [56, 57] and the downstream activation of the p130Cas/Src survival pathway [58]. We hypothesize that the high-affinity engagement of NRP1 by our hybrid may facilitate a ligand-induced receptor clustering shift. By favoring the assembly of the NRP1/PlexinA1 holoreceptor, the hybrid may effectively outcompete the recruitment of the GIPC1/adaptor complex, thereby silencing the p130Cas survival axis and initiating Plexin-mediated R-Ras inactivation [59] and the subsequent collapse of the actin cytoskeleton in endothelial cells. Furthermore, we intend to examine the phosphorylation status of VEGFR2 at the Y1175 site. This residue serves as the primary docking site for PLCγ1 and is an essential trigger for the ERK1/2 signaling cascade that drives endothelial proliferation [60]. Since NRP1 is known to specifically promote Y1175 phosphorylation by stabilizing the VEGFR2 signaling complex [46], we hypothesize that the SEMA-PSI-IG-HBD hybrid acts as a steric inhibitor. By decoupling the NRP1-VEGFR2 interaction, the hybrid should selectively suppress the proliferative signals required for the vascular disorganization and hyper-sprouting typically observed in U87-driven angiogenesis.

Third, and perhaps more importantly, the effects of SEMA-PSI-IG-HBD on angiogenesis and tumorigenesis must be examined in other *in vivo* systems, where its functional antagonism towards VEGF might show up and thus the design of the hybrid protein would justify our expectations. In addition, follow-up studies by analyzing gene expression changes in endothelial cells would provide further insights into how the hybrid SEMA3A-VEGF protein affects the angiogenesis process.

## Conclusions

In summary, the constructed SEMA-PSI-IG-HBD molecule acts as a potent antagonist of angiogenesis. Its ability to disrupt endothelial invasion and vessel development *in vitro* and *in ovo* identifies this hybrid structure as a viable candidate for further development in anti-angiogenic therapy.

## Supplementary Information

Below is the link to the electronic supplementary material.

Supplementary Material 1.

## Data Availability

The datasets used and/or analysed during the current study are available from the corresponding author on reasonable request.

## Abbreviations

CAM: Chicken embryo chorioallantoic membrane
DMEM: Dulbecco’s modified eagle medium
EDD: Day of embryo development
FBS: Fetal bovine serum
FHBD: Recombinant protein consisting of signal sequence of VEGF_165_ (aa 1–27), FLAG peptide and HBD domain of VEGF_165_ (aa 142–191)
HBD: Heparin-binding domain
HUVECs: Human umbilical vein endothelial cells
Ig-like: Immunoglobulin-like domain
LSGS: Low serum growth supplement
NRP_s_: Neuropilin receptors
P/s: Penicillin and streptomycin
PSI: Plexin-semaphorin-integrin motif
SD: Standard deviation
SEMA3A∆BD: Recombinant protein SEMA3A lacks the C-terminal basic domain
SEMA3s: Class 3 semaphorin proteins
SEMA-PSI-IG-HBD: Recombinant protein SEMA3A lacks the C`-terminal basic domain and directly fused to the HBD domain of VEGF
VEGF: Vascular endothelial growth factor
WCE: Whole-cell extract
