# Supplementary material for: Substitution of the SEMA3A basic domain with the VEGF165 heparin-binding domain enhances anti-angiogenic potency in ovo
Source: Biol Res. 2026 Mar 16;59:25. doi: 10.1186/s40659-026-00684-z (PMC13104550; doi:10.1186/s40659-026-00684-z)
Supplement: Supplementary file 1 — Supplementary Material 1. [file 40659_2026_684_MOESM1_ESM.pdf]

Additional file 1

Verification of constructed expression vectors

The 293 FT cells were transfected with expression vectors encoding SEMA3A, VEGF, SEMA-PSI-IG-HBD, SEMA3AΔBD, FHBD, and VENUS proteins. After 24 h, the fluorescence of protein VENUS was detected in SEMA3A, VEGF, SEMA-PSI-IG-HBD, FHBD, and VENUS groups, suggesting that the expression vectors were successfully delivered into the cells. Since the expression vector, encoding SEMA3AΔBD protein did not contain a sequence of a fluorescent protein VENUS, the fluorescence was not detected in these cells (Figure 1A). To verify, weather SEMA3AΔBD was synthetized in the 293 FT cells, reverse-transcription PCR was performed.

Total RNA was extracted from transfected cells with Trizol reagent (Invitrogen, 15596018), and cDNA was synthesized with a "High-Capacity cDNA Reverse Transcription Kit" (Applied Biosystems, 4368814), according to the manufacturer's instructions. The mRNA expression of SEMA3A, SEMA-PSI-IG-HBD, and SEMA3AΔBD were analyzed by reverse-transcription PCR. Reaction mix consisted of 6 µl of "Maxima Hot Start Green PCR Master Mix" (Thermo Scientific, K1062), 15 ng of cDNA, 0.2 µM of forward and reverse primers, and nuclease-free water to a final reaction volume of 12 µl. The reactions were performed at 95°C for 5 min, 30 cycles at 95°C for 20 s, 60°C for 30 s, 72°C for 2 min and 30 s, and final 72°C for 7 min. Amplification products were analyzed on a 0.8% agarose gel (Figure 1B).

Primer sequences used for the reaction are presented in Table 1.

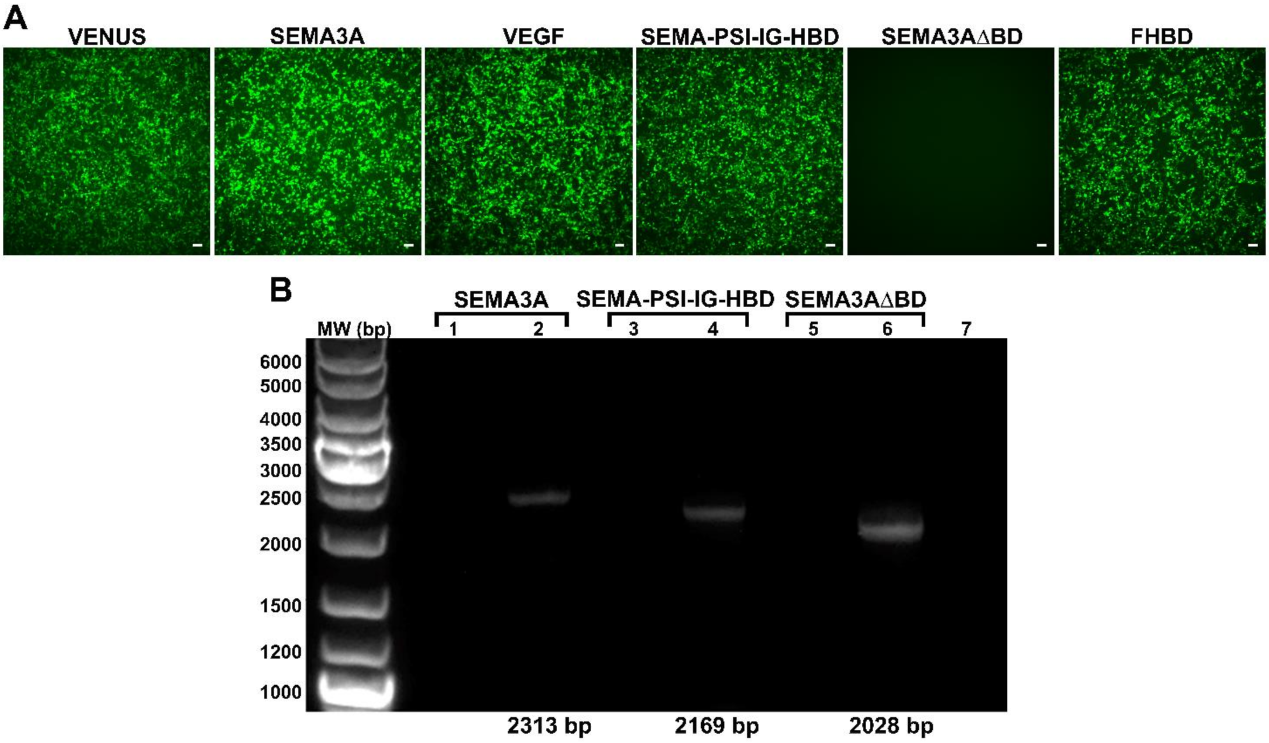

Figure 1. Verification of transfected 293 FT cells. (**A**) Venus-produced fluorescence in 293 FT cells transduced with expression vectors encoding VENUS, SEMA3A, VEGF, SEMA-PSI-IG-HBD, and SEMA3AΔBD proteins. Scale bar, 100 µm. (**B**) Expression analysis of SEMA3A, SEMA-PSI-IG-HBD, and SEMA3AΔBD by reverse-transcription PCR on 0.8% agarose gel. Lines 1, 3, and 5 – reactions with primers of the studied genes and cDNA obtained from 293 FT cells transfected with the control vector pTO/IRES2-VENUS; 2, 4, and 6 – reaction with primers of the studied genes and cDNA of cells transfected with vectors, encoding the studied proteins; 7 – control reaction with primers specific for the SEMA3A coding sequence and the cDNA sample without reverse transcriptase. MW – molecular weight of the ladder "GeneRuler DNA Ladder Mix" (Thermo Scientific, #SM0331).

Table 1. Primer sequences for reverse-transcription PCR.

Gene	Primers		Amplicon size, bp
	Forward 5`→ 3`	Reverse 5`→ 3`	
SEMA3A	TCGGATCCATGGGCTGG TTAACTAGGATTG	AGGGCACCCAGGAGTG TCTGAAGAAGATCTTT	2313
SEMA-PSI-IG-HBD	TCGGATCCATGGGCTGG TTAACTAGGATTG	GTGACAAGCCGAGGCG GTGAAGATCTAA	2169
SEMA3AΔBD	TCGGATCCATGGGCTGG TTAACTAGGATTG	GCCAGATCTTCATGTGT CAATGACTTCCAGGG	2028
